# New evidence for a hydroxylation pathway for anaerobic alkane degradation supported by analyses of functional genes and signature metabolites in oil reservoirs

**DOI:** 10.1186/s13568-020-01174-5

**Published:** 2021-01-12

**Authors:** Li-Bin Shou, Yi-Fan Liu, Jing Zhou, Zhong-Lin Liu, Lei Zhou, Jin-Feng Liu, Shi-Zhong Yang, Ji-Dong Gu, Bo-Zhong Mu

**Affiliations:** 1 State Key Laboratory of Bioreactor Engineering and School of Chemistry and Molecular Engineering, East China University of Science and Technology, 130 Meilong Road, Shanghai, 200237 People’s Republic of China; 2 Environmental Engineering, Guangdong Technion Israel Institute of Technology, 241 Daxue Road, Shantou, Guangdong 515063 People’s Republic of China; 3 Engineering Research Center of Microbial Enhanced Oil Recovery, East China University of Science and Technology, 130 Meilong Road, Shanghai, 200237 People’s Republic of China

**Keywords:** Alkane hydroxylation, Biodegradation, PCR detection, Signature biomarker, Primer design

## Abstract

Microbial degradation of recalcitrant alkanes under anaerobic conditions results in the accumulation of heavy oil fraction in oil reservoirs. Hydroxylation of alkanes is an important activation mechanism under anaerobic conditions, but the diversity and distribution of the responsible microorganisms in the subsurface environment are still unclear. The lack of functional gene polymerase chain reaction (PCR) primers and commercially available intermediate degradation chemical compounds are the major obstacles for this research. In this investigation, PCR primers for the *ahyA* gene (encoding alkane hydroxylase) were designed, evaluated, and improved based on the nucleotide sequences available. Using microbial genomic DNA extracted from oil-contaminated soil and production water samples of oil reservoirs, *ahyA* gene nucleotide sequences were amplified and retrieved successfully from production water sample Z3-25 of Shengli oilfield. Additionally, the signature biomarker of 2-acetylalkanoic acid was detected in both Shengli and Jiangsu oilfields. These results demonstrate that anaerobic hydroxylation is an active mechanism used by microorganisms to degrade alkanes in oxygen-depleted oil reservoirs. This finding expands the current knowledge of biochemical reactions about alkane degradation in subsurface ecosystems. In addition, the PCR primers designed and tested in this study serve as an effective molecular tool for detecting the microorganisms responsible for anaerobic hydroxylation of alkanes in this and other ecosystems.

## Key points

• PCR primers were designed and tested for detection of anaerobic alkane hydroxylation
• Anaerobic alkane hydroxylation was detected in the oil reservoirs for the first time
• Microbial hydroxylation products of hydroxylation were detected in oil reservoirs

## Introduction

Anaerobic degradation of hydrocarbon plays an important role in subsurface carbon cycling. Since anaerobic degradation of oil was first observed at an oil spill site in 1980 (Edwards and Wills 1992, Li et al. 2019), further evidence on anaerobic alkane degradation in a range of environments, including contaminated sites and oilfields has been reported widely (Agrawal and Gieg 2013; Bian et al. 2015). Crude oil is a complex mixture containing a wide range of chemical compounds with the main components being alkanes (Larter et al. 2003). For example, a recent study detected a high abundance of *n*-alkanes in the crude oil of Jiangsu oilfield (Bian 2015). The initial activation of alkanes, which involves the activation of thermodynamically stable C−C bonds of alkane molecules, is the critical and difficult step in anaerobic biodegradation of alkanes (Spormann and Widdel 2000, Liu et al. 2016a, 2016b). Currently, three mechanisms for oxygen-independent alkane C−H activation are known: fumarate addition (Callaghan et al. 2010), hydroxylation (Heider et al. 2016) and alkyl-Coenzyme M formation (Laso-Perez et al. 2016). Fumarate addition is thought to be the dominant activation mechanism in oil reservoirs (Agrawal and Gieg 2013; Mbadinga et al. 2011), which is supported by the detection of the *assA*/*bssA* genes and its degradation products alkyl-succinates in different oil reservoirs around the world (Agrawal et al. 2012; Bian et al. 2015; Duncan et al. 2009; Gieg et al. 2010). However, knowledge of alternative activation mechanisms, such as hydroxylation (Heider et al. 2016) and alkyl-Coenzyme M formation (Chen et al. 2019; Laso-Perez et al. 2016) remain extremely limited.

*Desulfococcus oleovorans* strain Hxd3, isolated from an oil–water separator of an oilfield, is the sole known alkane-degrading (C_12-20_) sulphate-reducing bacterium which utilises a fumarate-independent mechanism (Aeckersberg et al. 1991). It yields fatty acids with an even number of carbon atoms in the chain, if growing on an alkane with an odd number of carbon atoms. The same occurs with alkane with an even number of carbon atoms (Aeckersberg et al. 1998). This distinctive metabolite pattern is different from those produced by the fumarate addition mechanism. Furthermore, stable isotope studies indicate that the initial activation mechanism includes carboxylation at the C-3 position and then removal of two terminal carbon atoms (Ming et al. 2003). Proteomic analysis confirmed the absence of an alkyl-succinate synthase and the involvement of an ethylbenzene dehydrogenase ortholog, a putative alkane C2-methylene hydroxylase (AhyABC). Ahy was proposed to catalyze the anaerobic hydroxylation reaction at C2 followed by the oxidation of the hydroxy group to a keto group (Callaghan 2013), and the subsequent activation of C3 to be carboxylated (Rabus et al. 2016a; Rabus et al. 2016b). Recently, 2-acetylalkanonic acid was synthesised and proposed as a chemical biomarker of anaerobic *n*-alkane hydroxylation reaction (Zhou et al. 2016). However, this signature metabolite has not been detected and reported in any oil reservoir.

16S rRNA gene sequences affiliated with *D. oleovorans* have been detected repeatedly in oil contaminated samples (Abed et al. 2011; Acosta‐González et al. 2013; Davidova et al. 2007). This indicates a potential anaerobic hydrocarbon degradation via hydroxylation in hydrocarbon-associated environments. However, there is a substantial knowledge gap between the 16S rRNA gene repertoire and biochemical capabilities of a microorganism. Functional genes are considered to be diagnostic biomarkers for this potential biochemical reaction mechanism and suitable to detect the organisms which may not be dominant in the microbial population of a sample (Junier et al. 2010; Zhou et al. 2017).

Ethylbenzene dehydrogenase was first detected in several denitrifying bacterial strains closely related to *Rhodocyclaceae* species. This enzyme catalyses a hydroxylation reaction, in which a water molecule is incorporated into ethylbenzene to form (*S*)-1-phenylethanol. The catalytic enzyme has Mo(VI) as a co-factor. Genome sequencing of *D*. *oleovorans* Hxd3 revealed that an *ebdABC* ortholog gene, *ahyABC*, was involved (Callaghan 2013). Hence, strain Hxd3 degrades alkanes by a hydroxylation mechanism catalyzed by the AhyABC enzyme (Callaghan 2013). Several putative genes involved in the anaerobic alkane hydroxylation reaction have been detected in metagenomic studies of oil reservoir samples, indicating the potential metabolic capability of anaerobic alkane hydroxylation (Dong et al. 2019; Tan et al. 2015). However, metagenomic sequencing does not differentiate the biochemical functions of different microorganisms and no specific confirmation of the selective groups can be derived from environmental samples (Stepanauskas 2012). Therefore, a PCR-based approach targeting the specific functional genes for anaerobic alkane hydroxylation reaction will offer a specific and improved insight into the microbial diversity responsible for this biochemical process.

We designed several primers targeting the alpha subunit of anaerobic alkane hydroxylase (Ahy) and utilised these primers to detect the diversity of *ahyA* genes in oil reservoirs and oil contaminated soil samples. The potential anaerobic alkane degradation was further confirmed by detection of the chemical metabolite of the hydroxylation pathway. The results advance our knowledge on the alternative strategy of anaerobic alkane hydrocarbon degradation in addition to fumarate addition in oil reservoirs.

## Materials and methods

### Sampling and sites

Samples originated from production water and contaminated soils from two oil fields and a polluted mangrove wetland. Production water samples were collected from wells of Jiangsu and Shengli oilfields in China. These samples had a temperature of 63 ℃ and 80 ℃ at Shengli oilfield and Jiangsu oilfield, respectively. The water samples were collected into 10 L plastic containers, which were filled completely and then capped for transportation. At the same time, samples from oil contaminated soils were collected at Shengli and Jiangsu oilfields. In addition, coastal wetland sediment was collected from Mai Po Nature Reserve in Hong Kong. These soil and sediment samples were taken from subsurface layers, where oxygen was depleted and the concentrations of nitrate and sulfate were low (Hu et al. 2019). Soil and sediment samples were transferred into individual plastic bags and sealed for transportation and stored at − 20°C for preservation.

### Primer design

Nucleotide sequences of the *ahyA* gene (WP_012173623.1 and OQX63933.1) were aligned by software Mega7. A series of forward and reverse primers was designed according to these sequences. These primers were paired according to their positions and melting temperatures (T_m_). To test these initial primer pairs, a DNA template mix containing all of the environmental DNA was used initially. The primers were improved further based on the overlaps between the primer targets. The refined primer sets were tested further with the DNA template mix. Primer set 6’, which showed the highest diversity and specificity, was chosen for subsequent investigations on individual samples.

### DNA extraction and PCR amplification

Production water samples (1 L) were centrifuged at 12,000×*g* for 15 min at 4 ℃ to collect cell pellet. DNA was isolated from the biomass pellets using the AxyPrep™ Bacterial Genomic DNA Miniprep Kit (Axygen Biosciences, Inc., Union City, CA, USA) following the instructions of the manufacturers. For soil samples, DNA was extracted from 2 g of sediments using E.Z.N.A.^TM^ Soil DNA kit (D5625-01, Omega Bio-Tek, Inc., Norcross, GA, USA), according to its operating manual.

The 2× Taq PCR Master Mix (TaKaRa Bio, Shiga, Japan) was used to set up PCR reactions in 25 μL volume. Of all the primer sets designed in this study, primer set 6’ (ahyA1377_for_: 5′-AGCYTSGGCAAGAARGGMTGC-3′, ahyA1843’_rev_: 5′-ATGGTCTTRTAYTTDTY CCASAG-3′) was tested as the best one and then chosen to amplify the corresponding nucleotide sequences of this functional gene. PCR reaction conditions were: 98 ℃ for 1 min followed by 32 cycles of 98 ℃ for 15 s, 50 ℃ for 30 s, 72 ℃ for 40 s and a final extension step at 72 ℃ for 10 min. Subsequently, PCR products were pooled and purified with a DNA purification kit (Axygen Biosciences, Inc., Union City, CA, USA).

### Construction of ahyA gene clone libraries and phylogenetic analysis

Purified PCR products after electrophoresis were cloned into *Escherichia coli* DH5α using the pMD19^®^-T simple cloning vector (TaKaRa Bio) following the instructions of the manufacturer. Clones were cultured overnight in LB medium at 37 °C and screened using blue/white selection. To evaluate the performance of the primers, 30 clones were picked and sequenced. For each sample, subsequently 100 clones were picked and sequenced. The obtained sequences were first trimmed to eliminate vector and primer sequences before translating to corresponding amino acid sequences using the ORF Finder analysis tool. After clustering into operational taxonomic units (OTUs) having ≥ 97% sequence identity, using CD-HIT Suite (http://weizhongli-lab.org/cd-hit/), the representative OTUs were aligned with all known *ahyA* genes and other similar genes available publicly (accessed on 10th November, 2020). A phylogenetic tree was constructed in MEGA7 using the neighbour-joining method with the Poisson correction method and 1,000 bootstrap replicates.

### Detection of degradation intermediates

Detection of the degradation intermediates was carried out using a method described previously by this laboratory (Bian et al. 2015). The potential products were extracted from samples and derivatized via ethyl esterification. 500 mL of production water sample was placed into a 1 L flask with 4 g of KOH. The mixture was refluxed with stirring at 100 ℃ for 2 h. The aqueous phase was collected and extracted with 50 mL of *n*-hexane. The oil phase was mixed with 100 mL of 1% NaOH and the aqueous phase was combined with the extract mentioned previously. After it had been concentrated to 100 mL, the aqueous phase was adjusted to pH < 1 with HCl. The organic phase was extracted three times with 10 mL of ethyl acetate. Na_2_SO_4_ was added to remove the residual water. After that the solvent was removed by rotary evaporation to dryness. Then, 10 mL of ethanol, 10 mL of cyclohexane and 0.2 g of NaHSO_4_ were added. The flask equipped with a water separator was refluxed at 80 ℃ until no more water was produced. After cooling to room temperature, ethanol and cyclohexane were removed, and deionised water was added. Esters were extracted with 10 mL of ethyl acetate three times, and then combined. The ethyl acetate was removed after drying over anhydrous Na_2_SO_4_. The solution was filtered through a membrane filter (0.4-μm-pore size) prior to injection for analysis.

For the soil and sediment samples, 500 mL double distilled water were added and the suspension was transferred into a 1 L flask with 4 g of KOH. The contents were mixed thoroughly with a mechanical stirrer at 100 ℃ for 2 h. The mixture was filtered to remove the solids. The filtrate was treated further identically to the production water samples as described above. The water phase was separated, concentrated, acidified and extracted with 10 mL ethyl acetate (3 times). After removing the ethyl acetate solvent by rotary evaporation, the obtained fatty acids were mixed with 10 mL of ethanol, 10 mL of cyclohexane and 0.2 g of NaHSO_4_ and placed in a flask for reaction at 80 ℃ until no more water was produced. Then, the organic solvent was removed before the addition of deionized water followed by extraction with ethyl acetate for 3 times.

Gas chromatography (GC, Agilent 6890) equipped with an HP-5MS capillary column (30 m × 0.25 mm × 0.25 μm) and a mass detector (MSD, Agilent 5975) was used. The detection method used an auxiliary temperature of 280°C and the ion source temperature was held at 230 ℃. For long-chain fatty acids, the injection port temperature was held at 280 ℃. The oven temperature was held initially at 70 ℃ for 2 min, then increased to 280 ℃ at a rate of 10 ℃/min, and kept at this temperature for 30 min. Diagnostic ion fragments of 2-acetylalkanonic acid can be found at m/z 130, 115, 101 and [M-42]^+^ (Zhou et al. 2016).

### Accession numbers of sequences retrieved in this study

The nucleotide sequences of the *ahy*A gene-PCR-amplified in this study were assigned the accession numbers MN563800–MN564237.

## Results

### Physicochemical characteristics of water samples

The physicochemical characteristics of the production water samples are listed in Table 1. These samples were neutral or weakly acidic with pH ranging from 6.4 to 7.0. The concentration of Na^+^ ions ranged from 65.1 mM to 228.7 mM, and that of Cl^−^ from 106.9 mM to 144.5 mM. NO_3_^−^ was not detectable in samples Z3-13 and Z3-25. It had the highest concentration of 0.3 mM in sample H88-3. The level of SO_4_^2−^ was low (0.3 mM) in the Shengli oilfield and ranged from 22.4 to 31.0 mM in Jiangsu oilfield. H88-3 was the only sample, in which PO_4_^3−^ was detected.

**Table 1:** Production water samples used for *ahy*A genes testing

Samples	Shengli oil field			Jiangsu oil field		
	Z3-13	Z3-24	Z3-25	H88-3	H88-21	H88-30
Temperature (°C)	63	63	63	80	80	80
pH	7.0	6.4	6.7	6.7	6.7	6.7
Na^+^ (mM)	166	65.14	170	210	224	229
Mg^2+^ (mM)	1.56	1.50	1.98	0.22	0.19	0.24
Ca^2+^ (mM)	1.80	2.11	2.25	0.05	0.05	0.03
NH_4_^+^ (mM)	2.90	11.26	1.61	1.19	1.56	1.47
K^+^ (mM)	0.33	0.53	0.53	16.17	13.66	15.22
Cl^−^ (mM)	106	129	109	134	144	136
NO_3_^−^ (mM)	ND	0.14	ND	0.32	0.34	0.31
SO_4_^2−^ (mM)	0.03	0.03	0.03	23.03	22.41	31.00
PO_4_^3−^ (mM)	ND	ND	ND	16.64	ND	ND
CO_3_^2−^ (mM)	17.96	8.05	14.83	0.40	0.51	0.51
Formate (mM)	0.54	ND	0.052	ND	ND	0.01
Acetate (mM)	ND	ND	0.04	0.49	0.68	0.86
Propionate (mM)	0.05	ND	ND	ND	ND	ND
Butyrate (mM)	0.17	0.16	0.22	ND	ND	0.02

ND, not detected. The detection limit of formate and PO_4_^3−^ is 1×10^−3^ mM. The detection limit of acetate, propionate and butyrate is 2×10^−3^ mM

### Primers targeting putative ahy genes

**Fig. 1:**
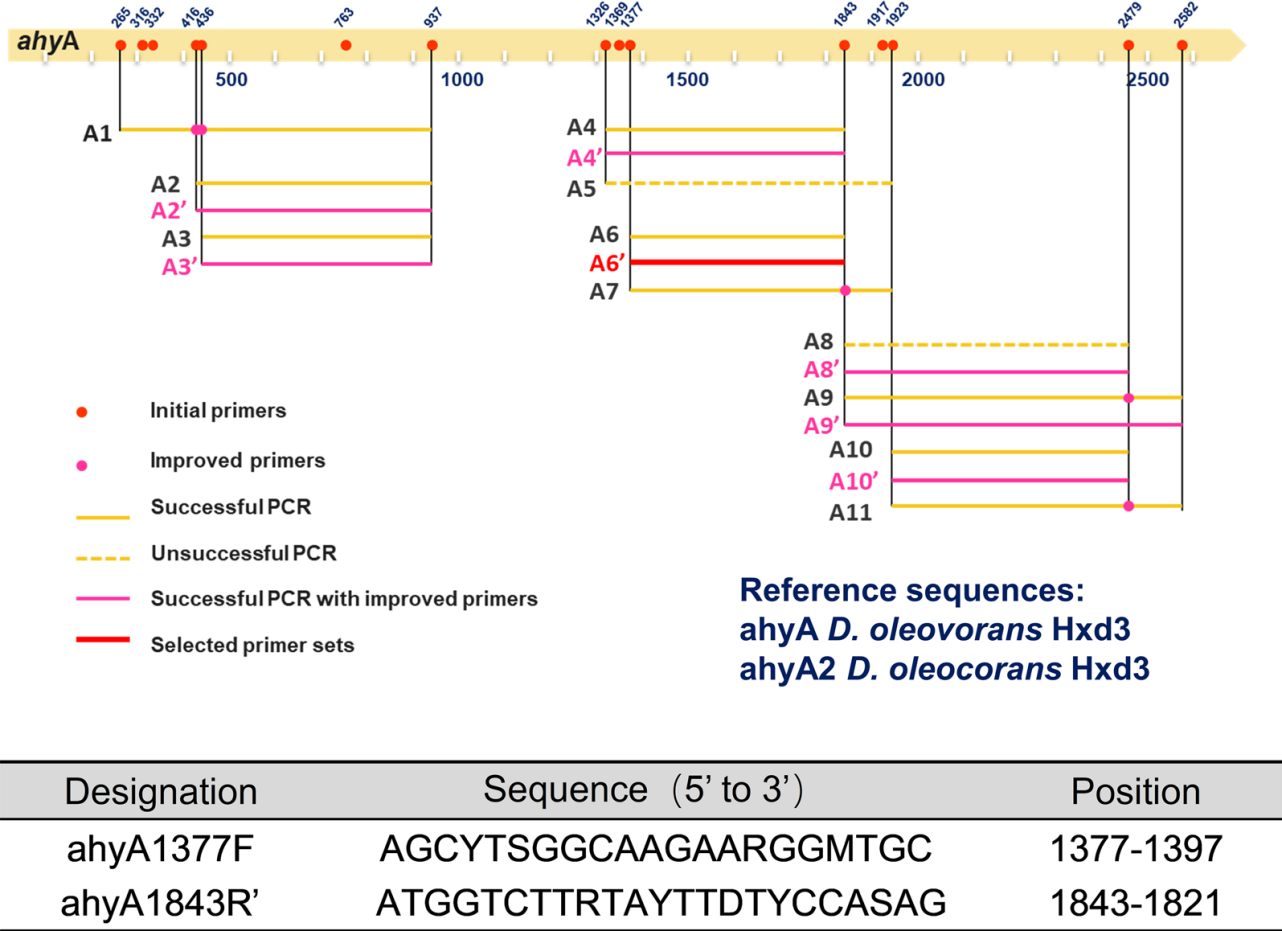
Location of the primers designed in this study with reference to *ahy* genes of *D. oleovorans*. The positioning of *ahy* primers refers to the *Desulfococcus oleovorans* strain Hxd3 *ahy* gene (WP_012173623.1). Initial primers are shown in red and improved primers are shown in pink. After being tested, primers ahyA1377F and ahyA1843R’ shown in red were selected for subsequent use

The *ahy* gene belongs to the subfamily 2 of the dimethyl sulfoxide reductase-type molybdenum enzymes. This subfamily includes ethylbenzene dehydrogenase (EBDH), which catalyzes the oxygen-independent hydroxylation of ethylbenzene. Eleven PCR primer sets were designed based on the two available *ahy* genes from the genome of the sulphate-reducing bacterium *D. oleovorans* (Fig. 1, accession numbers: OQX63933.1 and WP_012173623.1; Additional file 1: Table S1). Using the DNA mixture as templates, nucleotide sequences of the *ahy*A gene were amplified successfully with nine primer sets (Additional file 1: Table S2). For each set of primers, 30 clones were picked and sequenced. Sequence information of the amplicons generated with the first round of primers was then used to optimize the specificity of the second round of primers. Of these, primer set ahyA6’ displayed the highest specificity (four OTUs in 30 seqs, 100% specificity) (Additional file 1: Table S2) and was selected to recover putative *ahyA* gene sequences from different samples of oil reservoirs, soil and mangrove wetland. Detailed information about these pairs of PCR primes is provided in Additional file 1: Table S1.

### Phylogenetic analysis

**Fig. 2:**
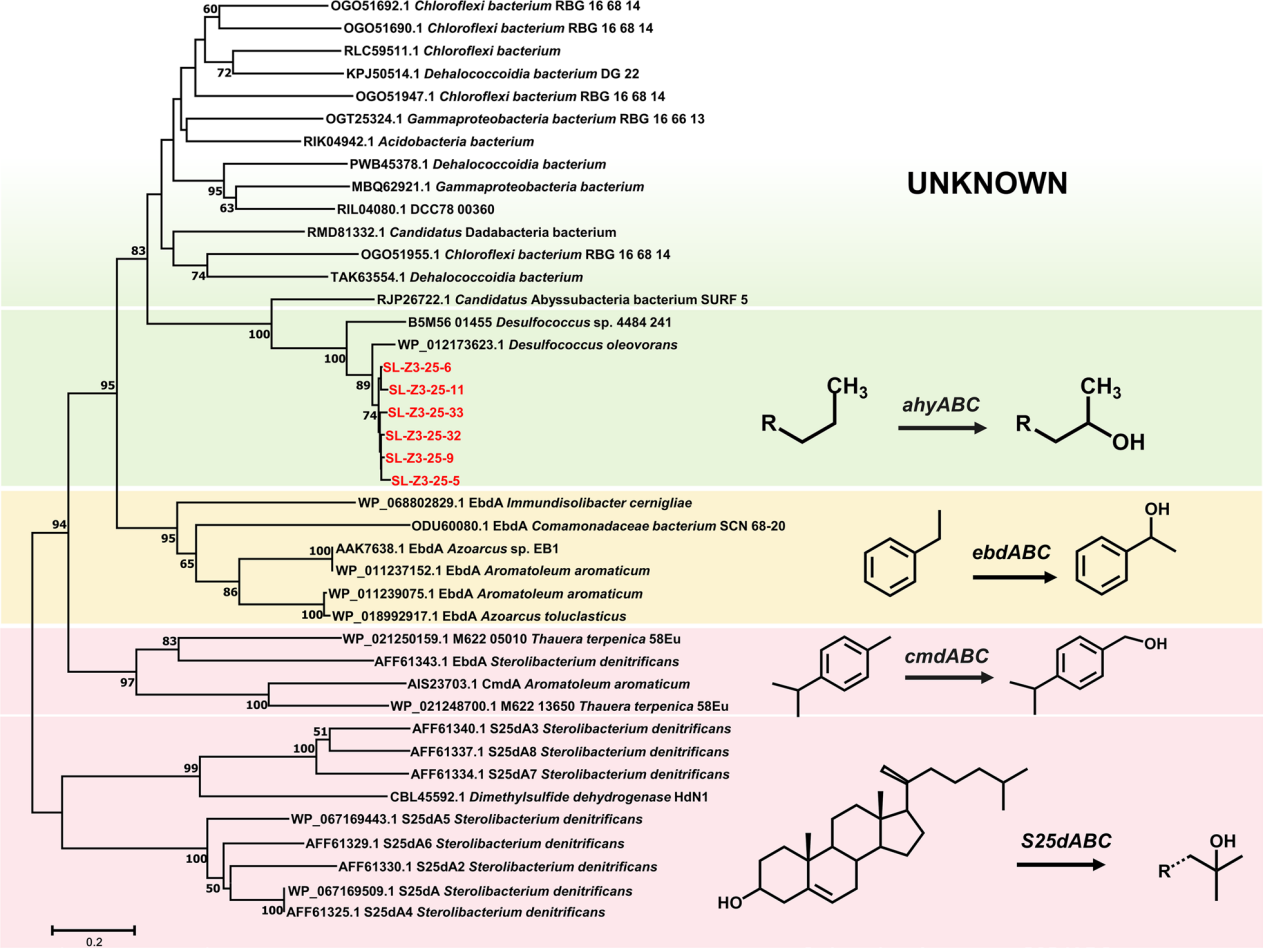
Bootstrapped neighbour-joining phylogeny of *ahyA* and selected Mo-dependent hydroxylase gene sequences (outgroup not shown here). The sequences retrieved in this study are shown in red. Bootstrap support (1,000 replicates) > 50% is indicated at the nodes. The scale bar represents 20% amino acid sequence divergence

Putative *ahy*A gene (alkane hydroxylation) fragment sequences were retrieved from the production water sample Z3-25 of Shengli oil reservoir. No sequences were retrieved from other samples of production water, polluted soils or wetland sediment. In total 100 sequences were obtained. They clustered into six OTUs (>97%) (Fig. 2). These sequences show a high amino acid identity (89–93%) to the *ahy*A sequence in strain Hxd3 (WP_012173623.1), suggesting that the primers have a high specificity. The retrieved sequences, reference sequences and other Mo-dependent hydrocarbon-degrading hydroxylase sequences (like p-cymene dehydrogenase and steroid C25 hydroxylase) (Heider et al. 2016) were used for the construction of a phylogenetic tree. For this purpose, sequences similar to the retrieved sequences, which were available from public database, were used. The phylogenetic analysis demonstrated that these sequences clustered within the alkane degradation group (Fig. 2), indicating that they may be involved in the catalysis of the anaerobic hydroxylation of alkanes.

### Characteristics of retrieved ahy sequences

The structure of EBDH from *A. aromaticum* strain EbN1 was acquired by X-ray crystallography. The active centre of EBDH is a molybdenum ion coordinated with six ligands (Kloer et al. 2006). In addition to the *cis*-dithiolene sulphur atoms of the two MGD molecules, Mo is ligated by the oxygen atoms of a bound acetate ion and an Asp residue (Asp223) connected by a Lys residue (Lys450). His192 is expected to function as a base to meet the strong pH dependence of the reaction. We analysed these conserved residues in our sequences. Unfortunately, site Lys450 was not detectable in our environmental *ahy*A sequences (Additional file 1: Fig. S1). Asp223 was present in all retrieved *ahy* sequences, while His192 was replaced by proline in all *ahy* sequences.

### Signature metabolite for anaerobic hydroxylation

**Fig. 3:**
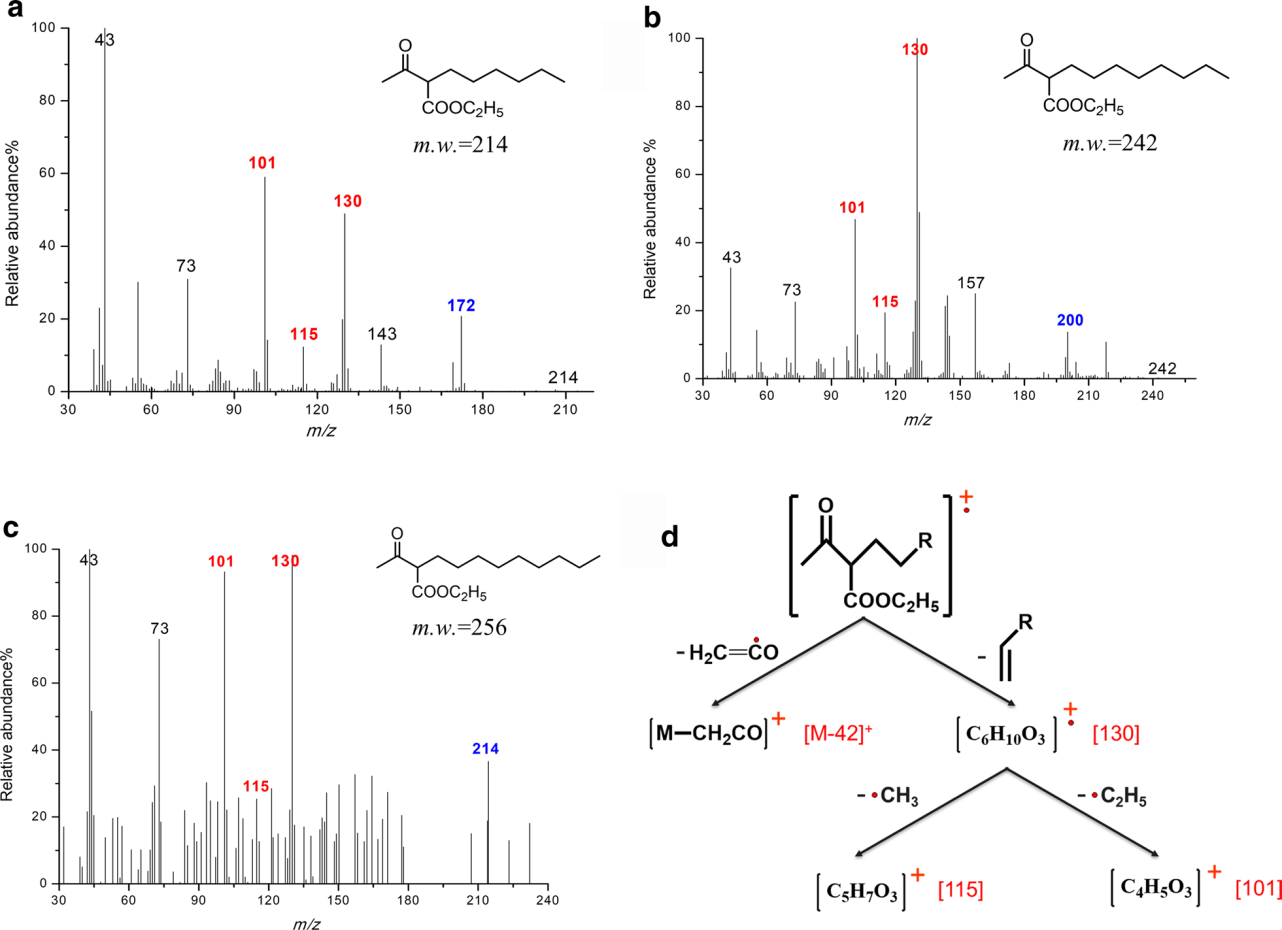
2-Acetylalkanoic acids as a signature metabolic intermediate in anaerobic alkane hydroxylation. Fragment ions at m/z 130, 115, 101 are shown in red, [M-42] is shown in blue. **a**, 2-Acetylnonic acid detected in H88-21; **b**, 2-Acetyl undecanoic acid detected in Z3-25; **c**, 2-Acetyl dodecanoic acid detected in Z3-13; **d**, characteristic fragments of ethyl 2-acetylalkanoic acids

2-Acetylalkanoic acids were used as signature metabolites for anaerobic hydroxylation of *n*-alkanes, because of their unique and specific structure among possible intermediates (Zhou et al. 2016). To search for these signature metabolites, six production water samples from two different oilfields, 2 polluted soils and a mangrove sediment were analysed using GC-MS. Fragment ions at *m/z* 101, 115 and 130 were used as indicators of 2-acetylalkanoic acid. 2-Acetylalkanoic acids derived from the parent *n*-alkanes with a chain length from C9 to C12 were detected successfully in samples Z3-13, Z3-25 and H88-21 (Fig. 3).

## Discussion

### Detection of environmental ahy genes and signature metabolites

Alkane hydroxylase (*ahy*ABC) catalyses the anaerobic hydroxylation of *n*-alkanes. Whether this biochemical progress is restricted to *D. oleovorans* strain Hxd3, is still unknown. There is no information about the environmental distribution of these genes. Currently, only a very limited number of *ahyA* gene nucleotide sequences are available in the public database (NCBI nr-nt), which has resulted in major limitations of the development of gene targeted detection assays. There is also a lack of knowledge about the distribution of *ahyA* genes in the microorganisms in different environments with variable community composition. To our knowledge, this is the first known attempt to design PCR primers targeting the *ahyA* gene with one of the reference sequences from the genome of *D. oleovorans* strain Hxd3. The other result came from a sulphate-reducing metagenome-assembled genome assigned to the *Desulfococcus* lineage (Dombrowski et al. 2017), showing an 84.7% identity (AAI) with the first one. Target genes were successfully amplified by nine pairs of primer sets initially in this study. The primers were highly specific in retrieving relevant sequences and improved further. The six retrieved *ahyA* OTU sequences and the two *Desulfococcus*-like *ahyA* sequences form a phylogenetic cluster distinctively different from the *ebdhA*, *cmdA* and *S25dA* clusters (Fig. 2).

In production water sample Z3-25, both the functional *ahyA* gene and the signature metabolite 2-acetyl undecanoic acid, were detected. 2-Acetyl dodecanoic acid and 2-acetylnonic acid were also detected in production water samples Z3-13 and H88-21, but unfortunately *ahyA* gene sequences could not be amplified from the DNA of these two samples. This was probably due to the high diversity of *ahyA* genes in samples Z3-13 and H88-21, which may not be retrieved by the presently designed primers, especially if considering that only very limited reference sequences are available, which can be used for the design of the present primers.

### Diversity of environmental ahyA gene lineages

After cloning and sequencing, a phylogenetic tree was constructed. Surprisingly, most of the similar sequences from the public database were affiliated with the *ahyA* cluster, rather than with the other hydroxylase clusters. These sequences came from oil reservoirs, mines, anammox reactors, and ocean and river sediments (Anantharaman et al. 2016; Baker et al. 2015; Chen et al. 2018; Momper et al. 2017; Tully et al. 2018; Zhao et al. 2018), which are all anoxic and hydrocarbon-impacted environments. Interestingly, the sequences, which shared the highest identity with the retrieved sequences, excluding the reference sequences, were from a metagenome-assembled genome (MAG, SURF_5) belonging to a new candidate phylum designated as Abyssubacteria (Momper et al. 2017). SURF_5 was a near-complete MAG containing a putative nitric oxide reductase (*norBC*). Thus, it was hypothesized to use nitrate or nitric oxide as an electron acceptor. Although *D. oleovorans* is a sulphate-reducing bacterium, hydroxylation has been described in a nitrate reducing consortium (Callaghan et al. 2009). Conserved residues are one of the auxiliary criteria for judging the function of enzymes. Asp223 and Lys 450 were highly conserved in all these sequences, but His192 was replaced by various amino acids in putative *ahy* sequences due to the different substrates, which was also consistent in the *cmd* and *S25d* sequences. These results suggest that this type of Mo-dependent hydroxylase could be much more diverse genetically than we thought.

### Anaerobic hydroxylation of alkanes in oil reservoirs

**Fig. 4:**
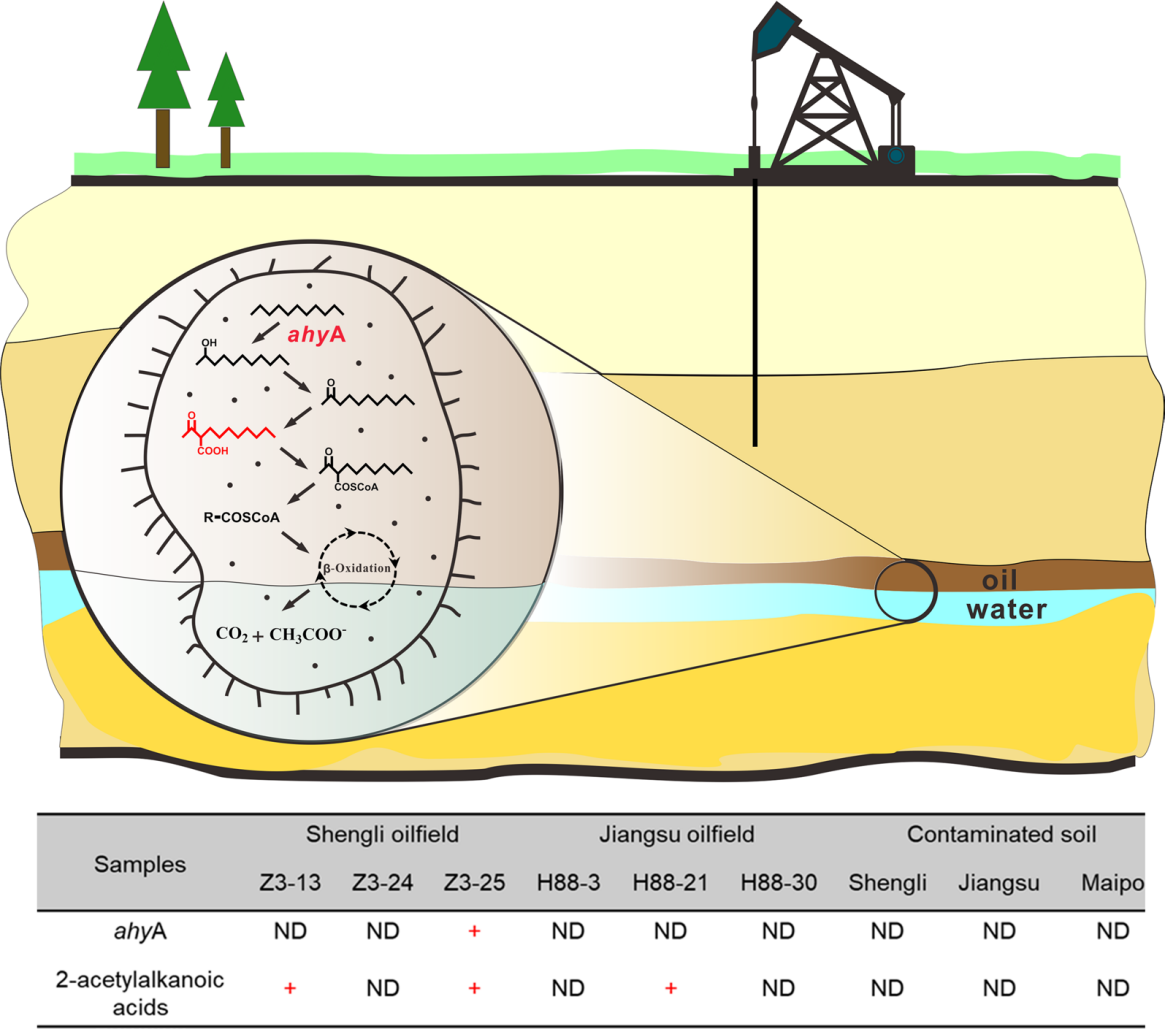
The signature metabolite and functional gene detected in samples. Red “+”: detected. Both *ahyA* and 2-acetylalkanoic acid are detected in production water sample Z3-25

Functional genes and the corresponding signature metabolites were detected simultaneously in the Z3-25 oil reservoir. The finding indicates that alkanes are activated via hydroxylation in this environment (Fig. 4). Oil reservoir environments are usually characterized as high temperature, high pressure, high salinity, and anoxic. Fumarate addition is the prominent biochemical activation mechanism for anaerobic alkane degradation in subsurface environments (Aitken et al. 2004). *n*-Alkanes are first initiated by the addition of fumarate at the subterminal or terminal (with propane) carbon atom. The reaction is catalyzed by the alkyl-succinate synthase (*ass*) (Callaghan et al. 2010). Further degradation involves carbon skeleton re-arrangement, decarboxylation, and *β*-oxidation. The initial products, 2-(1-methylalkyl) succinates, are generally regarded as biochemical markers. Alkyl-succinate and the *ass*A gene have been found in production water originating from oil reservoirs (Agrawal and Gieg 2013; Bian et al. 2015). However, the metabolites and functional genes of other initial activation reactions have never been reported. The contributions of hydroxylation for the degradation of alkanes and the niche of hydroxylation promoting bacteria are still unknown.

In the present study, primers of genes responsible for anaerobic alkane hydroxylation (*ahyA*) were designed, tested and optimized. Six samples from two oil reservoirs of production water and polluted soils and in addition one of a mangrove sediment were investigated for anaerobic alkane hydroxylation by an integrated method using both PCR and GC-MS. 2-Acetylalkanonic acids were detected in three of the six production water samples. In the putative anaerobic hydroxylation pathway, 2-Acetylalkanonic acids were produced after the hydroxylation and subsequent oxidation of alkanes by microorganisms. The 2-acetylalkanonic acids detected in this study were the potential intermediate metabolites during the degradation of *n*-nonane, *n*-undecane and *n*-dodecane, which are common compounds in crude oil (Larter et al. 2003). Simultaneously, *ahyA* was found in the sample Z3-25, where both the functional gene and a signature metabolite were detected. The findings of the combined method strongly support that anaerobic activation of alkanes via the alkane hydroxylation pathway occurs in oil reservoirs.

## Supplementary Information

**Additional file 1: Fig. S1.** Multiple-sequence alignment of selected putative EBDH and AHY enzymes around the catalytic site of EBDH. Representative environmental Ahy enzymes were deduced from the gene sequences generated in this study. The conserved residues for catalytic functioning in EBDH are highlighted in red, whereas substitutions at these sites in putative AHY are shown in blue. **Table S1.** Primers designed in this study (len: length, pos: start position, gc: GC content; Tm: melting temperature). **Table S2.** Performance of primers

## Data Availability

Raw reads are available in the GenBank archive at the National Center for Biotechnological Information (NCBI) as listed in the manuscript.

## Abbreviations

ahyA: Anaerobic alkane hydroxylase gene
ebdA: Ethylbenzene dehydrogenase gene

## References

[CR1] Abed RMM, Musat N, Musat F (2011). Structure of microbial communities and hydrocarbon-dependent sulfate reduction in the anoxic layer of a polluted microbial mat. Mar Pollut Bull.

[CR2] Acosta-González A, Rosselló-Móra R, Marqués S (2013). Characterization of the anaerobic microbial community in oil-polluted subtidal sediments: aromatic biodegradation potential after the Prestige oil spill. Environ Microbiol.

[CR3] Aeckersberg F, Bak F, Widdel F (1991). Anaerobic oxidation of saturated hydrocarbons to CO_2_ by a new type of sulfate-reducing bacterium. Arch Microbiol.

[CR4] Aeckersberg F, Rainey FA, Widdel F (1998). Growth, natural relationships, cellular fatty acids and metabolic adaptation of sulfate-reducing bacteria that utilize long-chain alkanes under anoxic conditions. Arch Microbiol.

[CR5] Agrawal A, Gieg LM (2013). In situ detection of anaerobic alkane metabolites in subsurface environments. Front Microbiol.

[CR6] Agrawal A, Park HS, Nathoo S, Gieg LM, Jack TR, Miner K, Ertmoed R, Benko A, Voordouw G (2012). Toluene depletion in produced oil contributes to souring control in a field subjected to nitrate injection. Environ Sci Technol.

[CR7] Aitken CM, Jones DM, Larter SR (2004). Anaerobic hydrocarbon biodegradation in deep subsurface oil reservoirs. Nature.

[CR8] Anantharaman K, Brown CT, Hug LA, Sharon I, Castelle CJ, Probst AJ, Thomas BC, Singh A, Wilkins MJ, Karaoz U (2016). Thousands of microbial genomes shed light on interconnected biogeochemical processes in an aquifer system. Nat Commun.

[CR9] Baker BJ, Lazar CS, Teske AP, Dick GJ (2015). Genomic resolution of linkages in carbon, nitrogen, and sulfur cycling among widespread estuary sediment bacteria. Microbiome.

[CR10] Bian XY, Mbadinga SM, Liu YF, Yang SZ, Liu JF, Ye RQ, Gu JD, Mu BZ (2015). Insights into the anaerobic biodegradation pathway of n-alkanes in oil reservoirs by detection of signature metabolites. Sci Rep.

[CR11] Bian XY (2015) The research of anaerobic biodegradation mechanism of petroleum hydrocarbons in oil reserviors. Dissertation, East China University of Science and Technology

[CR12] Callaghan AV (2013). Enzymes involved in the anaerobic oxidation of n-alkanes: from methane to long-chain paraffins. Front Microbiol.

[CR13] Callaghan AV, Davidova IA, Kristen SA, Parisi VA, Gieg LM, Suflita JM, Kukor JJ, Boris W (2010). Diversity of benzyl- and alkylsuccinate synthase genes in hydrocarbon-impacted environments and enrichment cultures. Environ Sci Technol.

[CR14] Callaghan AV, Meghan T, Phelps CD, Young LY (2009). Anaerobic biodegradation of n-hexadecane by a nitrate-reducing consortium. Appl Environ Microbiol.

[CR15] Chen C, Leu AO, Xie GJ, Guo J, Feng Y, Zhao JX, Tyson GW, Yuan Z, Hu S (2018). A methanotrophic archaeon couples anaerobic oxidation of methane to Fe(III) reduction. ISME J.

[CR16] Chen SC, Musat N, Lechtenfeld OJ, Paschke H, Schmidt M, Said N, Popp D, Calabrese F, Stryhanyuk H, Jaekel U, Zhu Y-G, Joye SB, Richnow H-H, Widdel F, Musat F (2019). Anaerobic oxidation of ethane by archaea from a marine hydrocarbon seep. Nature.

[CR17] Davidova IA, Gieg LM, Duncan KE, Suflita JM (2007). Anaerobic phenanthrene mineralization by a carboxylating sulfate-reducing bacterial enrichment. ISME J.

[CR18] Dombrowski N, Seitz KW, Teske AP, Baker BJ (2017). Genomic insights into potential interdependencies in microbial hydrocarbon and nutrient cycling in hydrothermal sediments. Microbiome.

[CR19] Dong XY, Greening C, Rattray JE, Chakraborty A, Hubert CRJ (2019). Metabolic potential of uncultured bacteria and archaea associated with petroleum seepage in deep-sea sediments. Nat Commun.

[CR20] Duncan KE, Gieg LM, Parisi VA, Tanner RS, Tringe SG, Bristow J, Suflita JM (2009). Biocorrosive thermophilic microbial communities in alaskan north slope oil facilities. Environ Sci Technol.

[CR21] Edwards EA, Wills LE (1992). Anaerobic degradation of toluene and xylene by aquifer microorganisms under sulfate-reducing conditions. Appl Environ Microbiol.

[CR22] Gieg LM, Davidova IA, Duncan KE, Suflita JM (2010). Methanogenesis, sulfate reduction and crude oil biodegradation in hot Alaskan oilfields. Environ Microbiol.

[CR23] Heider J, Szaleniec M, Suenwoldt K, Boll M (2016). Ethylbenzene dehydrogenase and related molybdenum enzymes involved in oxygen-independent alkyl chain hydroxylation. J Mol Microbiol Biotechnol.

[CR24] Hu QQ, Zhou ZC, Liu YF, Zhou L, Mbadinga SM, Liu JF, Yang SZ, Gu JD, Mu BZ (2019). High microbial diversity of the nitric oxide dismutation reaction revealed by PCR amplification and analysis of the nod gene. Int Biodeterior Biodegrad.

[CR25] Junier P, Molina V, Dorador C, Hadas O, Kim O-S, Junier T, Witzel K-P, Imhoff JF (2010). Phylogenetic and functional marker genes to study ammonia-oxidizing microorganisms (AOM) in the environment. Appl Microbiol Biotechnol.

[CR26] Kloer DP, Hagel C, Heider J, Schulz GE (2006). Crystal structure of ethylbenzene dehydrogenase from aromatoleum aromaticum. Structure.

[CR27] Larter S, Wilhelms A, Head I, Koopmans M, Aplin A, Di Primio R, Zwach C, Erdmann M, Telnaes N (2003). The controls on the composition of biodegraded oils in the deep subsurface—part 1: biodegradation rates in petroleum reservoirs. Org Geochem.

[CR28] Laso-Perez R, Wegener G, Knittel K, Widdel F, Harding KJ, Krukenberg V, Meier DV, Richter M, Tegetmeyer HE, Riedel D, Richnow HH, Adrian L, Reemtsma T, Lechtenfeld OJ, Musat F (2016). Thermophilic archaea activate butane via alkyl-coenzyme M formation. Nature.

[CR29] Li D-S, Feng J-Q, Liu Y-F, Zhou L, Liu J-F, Gu J-D, Mu B-Z, Yang S-Z (2019). Enrichment and immobilization of oil-degrading microbial consortium on different sorbents for bioremediation testing under simulated aquatic and soil conditions. Appl Environ Biotechnol.

[CR30] Liu J-F, Mbadinga SM, Ke W-J, Gu J-D, Mu B-Z (2016). The diversity of hydrogen-producing microorganisms in a high temperature oil reservoir and its potential role in promoting the *in situ* bioprocess. Appl Environ Biotechnol.

[CR31] Liu J-F, Wu W-L, Yao F, Wang B, Zhang B-L, Mbadinga SM, Gu J-D, Mu B-Z (2016). A thermophilic nitrate-reducing bacterium isolated from production water of a high temperature oil reservoir and its inhibition on sulfate-reducing bacteria. Appl Environ Biotechnol.

[CR32] Mbadinga SM, Wang L-Y, Zhou L, Liu J-F, Gu J-D, Mu B-Z (2011). Microbial communities involved in anaerobic degradation of alkanes. Int Biodeterior Biodegrad.

[CR33] Ming SC, Phelps CD, Young LY (2003). Anaerobic transformation of alkanes to fatty acids by a sulfate-reducing bacterium, strain Hxd3. Appl Environ Microbiol.

[CR34] Momper L, Jungbluth SP, Lee MD, Amend JP (2017). Energy and carbon metabolisms in a deep terrestrial subsurface fluid microbial community. ISME J.

[CR35] Rabus R, Boll M, Golding B, Wilkes H (2016). Anaerobic degradation of p-Alkylated benzoates and toluenes. J Mol Microbiol Biotechnol.

[CR36] Rabus R, Boll M, Heider J, Meckenstock RU, Buckel W, Einsle O, Ermler U, Golding BT, Gunsalus RP, Kroneck PM (2016). Anaerobic microbial degradation of hydrocarbons: from enzymatic reactions to the environment. J Mol Microbiol Biotechnol.

[CR37] Spormann AM, Widdel F (2000). Metabolism of alkylbenzenes, alkanes, and other hydrocarbons in anaerobic bacteria. Biodegradation.

[CR38] Stepanauskas R (2012). Single cell genomics: an individual look at microbes. Curr Opin Microbiol.

[CR39] Tan B, Fowler SJ, Abu LN, Dong X, Sensen CW, Foght J, Gieg LM (2015). Comparative analysis of metagenomes from three methanogenic hydrocarbon-degrading enrichment cultures with 41 environmental samples. ISME J.

[CR40] Tully BJ, Graham ED, Heidelberg JF (2018). The reconstruction of 2,631 draft metagenome-assembled genomes from the global oceans. Sci Data.

[CR41] Zhao Y, Liu S, Jiang B, Feng Y, Zhu T, Tao H, Tang X, Liu S (2018). Genome-centered metagenomics analysis reveals the symbiotic organisms possessing ability to cross-feed with anammox bacteria in anammox consortia. Environ Sci Technol.

[CR42] Zhou J, Bian XY, Zhou L, Mbadinga SM, Yang SZ, Liu JF, Gu JD, Mu BZ (2016). Synthesis and characterization of anaerobic degradation biomarkers of n-alkanes via hydroxylation/carboxylation pathways. Eur J Mass Spectrom.

[CR43] Zhou ZC, Chen J, Meng H, Dvornyk V, Gu J-D (2017). New PCR primers targeting hydrazine synthase and cytochromecbiogenesis proteins in anammox bacteria. Appl Microbiol Biotechnol.

