# Supplementary material for: New evidence for a hydroxylation pathway for anaerobic alkane degradation supported by analyses of functional genes and signature metabolites in oil reservoirs
Source: AMB Express. 2021 Jan 12;11:18. doi: 10.1186/s13568-020-01174-5 (PMC7803848; doi:10.1186/s13568-020-01174-5)
Supplement: Supplementary file 1 — Additional file 1: Fig. S1. Multiple-sequence alignment of selected putative EBDH and AHY enzymes around the catalytic site of EBDH. Representative environmental Ahy enzymes were deduced from the gene sequences generated in this study. The conserved residues for catalytic functioning in EBDH are highlighted in red, whereas substitutions at these sites in putative AHY are shown in blue. Table S1. Primers designed in this study (len: length, pos: start position, gc: GC content; Tm: melting temperature). Table S2. Performance of primers [file 13568_2020_1174_MOESM1_ESM.pdf]

Supplementary materials

AMB express

New evidence for a hydroxylation pathway for anaerobic alkane degradation supported by analyses of functional genes and signature metabolites in oil reservoirs

Li-Bin Shou[1, †], Yi-Fan Liu[1, †], Jing Zhou[1], Zhong-Lin Liu[1], Lei-Zhou[1], Jin-Feng Liu[1], Shi-Zhong Yang[1], Ji-Dong Gu[2], Bo-Zhong Mu[1, 3*]

[1]*State Key Laboratory of Bioreactor Engineering and School of Chemistry and Molecular Engineering, East China University of Science and Technology, 130 Meilong Road, Shanghai 200237, P.R. China*

[2] *Environmental Engineering, Guangdong Technion Israel Institute of Technology, 241 Daxue Road, Shantou, Guangdong 515063, P.R. China*

[3]*Engineering Research Center of Microbial Enhanced Oil Recovery, East China University of Science and Technology, 130 Meilong Road, Shanghai 200237, P.R. China*

Corresponding Authors:

Prof. Bo-Zhong Mu E-mail: bzmu@ecust.edu.cn

[†]These authors contributed equally to this work.

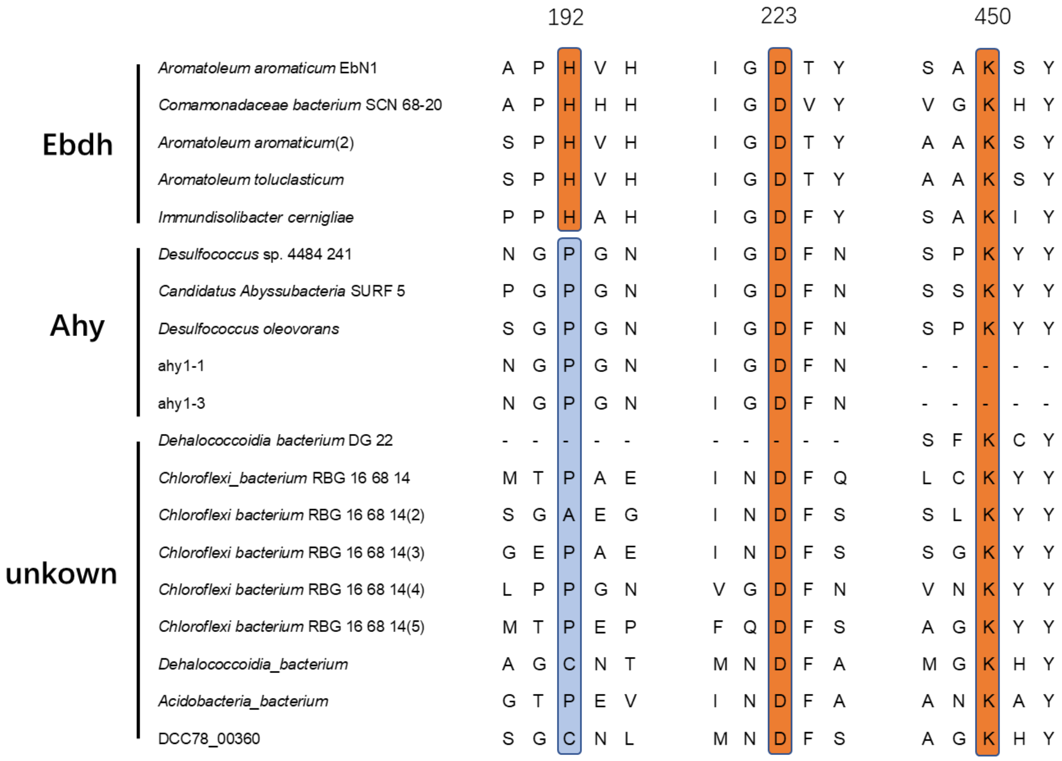

Fig. S1 Multiple-sequence alignment of selected putative EBDH and AHY enzymes around the catalytic site of EBDH. Representative environmental Ahy enzymes were deduced from the gene sequences generated in this study. The conserved residues for catalytic functioning in EBDH are highlighted in red, whereas substitutions at these sites in putative AHY are shown in blue.

Table. S1 Primers designed in this study (len: length, pos: start position, gc: GC content; Tm: melting temperature)

Name	Sequence	len	pos	gc	Tm
AhyA265F	GTGTTCMGKGAGGAGCAGG	19	265	0.632	58 ºC
AhyA316F	CCCGATTTYAAYCCCMGGG	18	316	0.605	57.2 ºC
AhyA332F	GGGGSTGCCAGAARGG	15	332	0.719	57.7 ºC
AhyA416F	GGGGMAGCGGCAAATGGAA	19	416	0.605	60.2 ºC
AhyA436F	CGGRTMAGCTGGGACCAGT	19	436	0.632	59.5 ºC
AhyA763F	TCMGAGGCCCGTTACAACG	19	763	0.605	58.6 ºC
AhyA937F	GAGCARAGCGACCTKCCCCT	20	937	0.65	62.2 ºC
AhyA937R	AGGGGMAGGTCGCTYTGCTC	20	937	0.65	62.2 ºC
AhyA1326F	TCACGGCGACCTGATTGARCGK	21	1326	0.591	62.5 ºC
AhyA1369R	TCTTGCCSARRCTSCCSGTAAG	22	1369	0.591	61.5 ºC
AhyA1377R	GCATCCYTTCTTGCCSARRCT	21	1377	0.548	59.1 ºC
AhyA1377F	AGCYTSGGCAAGAARGGMTGC	21	1377	0.595	61.7 ºC
AhyA1843R	ATGGTCTTGTAYTTWTYCCASAG	23	1843	0.391	52.6 ºC
AhyA1923R	CTTTTCRTARAAMCCGGCRCA	21	1923	0.476	56.1 ºC
AhyA1917R	GCTCAKGGCAGGGCATARTC	20	1917	0.6	58.4 ºC
AhyA1843F	CTGTGGRAWAARTACAAGACCAT	23	1843	0.391	52.8 ºC
AhyA1923F	TGTGCCGGKTTYTAYGAAAAGC	21	1923	0.477	57.4 ºC
AhyA1917F	GACTATGCCCTGCCMTGYGC	20	1917	0.65	61.3 ºC
AhyA2479R	CTCCAGGAACCASGGATGATC	21	2479	0.571	57.2 ºC
AhyA2582R	CCAGATGGAATGGAYGCTCCACC	23	2582	0.587	60.9 ºC
ahyA416F’	DGGGMAGCGGCAAATGGAA	19	416	0.605	60.2 ºC
ahyA436F’	CGGRYMAGCTGGRACCAGT	19	436	0.632	59.5 ºC
AhyA1843F’	CTGTGGRAHAARTAYAAGACCAT	23	1843	0.391	52.8 ºC
AhyA1843R’	ATGGTCTTRTAYTTDTYCCASAG	23	1843	0.391	52.6 ºC
AhyA2479R’	CTCCAGGAACCASGGATGRTC	21	2479	0.571	57.2 ºC

Table. S2 Performance of primers

Set	Forward primer	Reverse primer	OTUs (97%) in 30 clones	Specificity
1	AhyA265F	AhyA937R	2	100%
2	AhyA416F	AhyA937R	3	100%
3	AhyA436F	AhyA937R	2	100%
4	AhyA1326F	AhyA1843R	1	100%
5	AhyA1326F	AhyA1923R	0	100%
6	AhyA1377F	AhyA1843R	5	100%
7	AhyA1377F	AhyA1923R	1	100%
8	AhyA1843F	AhyA2479R	0	100%
9	AhyA1843F	AhyA2582R	1	100%
10	AhyA1923F	AhyA2479R	2	100%
11	AhyA1923F	AhyA2582R	1	100%
2’	AhyA416F’	AhyA937R	3	100%
3’	AhyA436F’	AhyA937R	3	100%
4’	AhyA1326F	AhyA1843R’	2	100%
6’	AhyA1377F	AhyA1843R’	5	100%
8’	AhyA1843F’	AhyA2479R	1	100%
9’	AhyA1843F’	AhyA2582R	2	100%
10’	AhyA1923F	AhyA2479R’	2	100%
